# The SR Protein B52/SRp55 Is Required for DNA Topoisomerase I Recruitment to Chromatin, mRNA Release and Transcription Shutdown

**DOI:** 10.1371/journal.pgen.1001124

**Published:** 2010-09-16

**Authors:** François Juge, Céline Fernando, Weronika Fic, Jamal Tazi

**Affiliations:** Institut de Génétique Moléculaire de Montpellier UMR 5535 CNRS, 1919 route de Mende, 34293 Montpellier cedex 5, France; Université Montpellier 2, Place Eugène Bataillon, 34095 Montpellier cedex 5; Université Montpellier 1, 5 Bd Henry IV, 34967 Montpellier cedex 2.; University of Washington, United States of America

## Abstract

DNA- and RNA-processing pathways are integrated and interconnected in the eukaryotic nucleus to allow efficient gene expression and to maintain genomic stability. The recruitment of DNA Topoisomerase I (Topo I), an enzyme controlling DNA supercoiling and acting as a specific kinase for the SR-protein family of splicing factors, to highly transcribed loci represents a mechanism by which transcription and processing can be coordinated and genomic instability avoided. Here we show that *Drosophila* Topo I associates with and phosphorylates the SR protein B52. Surprisingly, expression of a high-affinity binding site for B52 in transgenic flies restricted localization, not only of B52, but also of Topo I to this single transcription site, whereas B52 RNAi knockdown induced mis-localization of Topo I in the nucleolus. Impaired delivery of Topo I to a heat shock gene caused retention of the mRNA at its site of transcription and delayed gene deactivation after heat shock. Our data show that B52 delivers Topo I to RNA polymerase II-active chromatin loci and provide the first evidence that DNA topology and mRNA release can be coordinated to control gene expression.

## Author Summary

DNA Topoisomerase I (Topo I) is a very well known enzyme capable of removing DNA topological constrains during transcription. In mammals, Topo I also harbours an intrinsic protein kinase activity required to achieve specific phosphorylation of factors in charge of maturating the transcript and exporting it from the transcription site in the nucleus to the cytoplasm. In this report, we have used *Drosophila* genetics to describe the surprising finding that Topo I is not directly recruited to active transcription sites by DNA but rather by an indirect interaction with its protein target of phosphorylation which in turn is bound to nascent transcripts at gene loci. Furthermore, we demonstrate that the delivery of Topo I to an activated *heat shock* gene is essential for efficient release of the mRNA from its transcription site and functions to turn off transcription of the gene. This study brings a new model for the long unanswered question of how genes are turned off and provides evidence that Topo I is at the heart of the mechanism by which DNA and RNA processes are coordinately regulated during development to avoid genomic instability.

## Introduction

Messenger RNA (mRNA) transcribed by the RNA polymerase II (RNA Pol II) undergoes several maturation steps: capping, splicing and polyadenylation, before its export into the cytoplasm (for review see [1]). All these steps are tightly coupled to ongoing transcription so that RNA emerging from the polymerase is immediately coated with RNA-binding proteins that participate in RNA maturation, processing and assembly into an export-competent mRNA-ribonucleoprotein (mRNP) [2], [3]. Recent data show that transcriptional and post-transcriptional events mutually influence each other, revealing a reciprocal coupling. For example, transcription speed can influence splicing of the transcript, and factors involved in splicing of the emerging pre-mRNA can modulate transcription [1], [3]. Among the factors that have been proposed to play a role in the coupling between transcription and maturation of the pre-mRNAs is the DNA topoisomerase I (Topo I), a protein that carries two enzymatic activities: a ‘topoisomerase’ activity that relaxes DNA supercoiling generated by transcription, replication or chromatin dynamics and a ‘kinase’ activity that phosphorylates RNA splicing factors [4], [5].

Topo I is a type IB DNA topoisomerase that can relax both negative and positive supercoils during transcription and replication by introducing a single strand break into the DNA [6]. Although Topo I is not essential in yeast [6], [7], it is required for embryonic development in *Drosophila* [8] and mice [9]. A large part of our knowledge of Topo I activity has been obtained through the use of a highly specific drug, camptothecin, that traps the enzyme once it has cleaved the DNA [5]. This property allowed mapping of Topo I cleavage sites which are scattered along regions of DNA that are actively transcribed but it does not cleave the same regions when they are silent. An immediate effect of camptothecin is the inhibition of transcription [5] that occurs due to collision of stalled Topo I with transcribing RNA Pol II [10]. Through its action of relaxing the positive supercoils generated ahead of, as well as negative supercoils generated behind, transcribing RNA Pol II, it has been suggested that Topo I plays a major role in transcription [11]. However there is no direct evidence that the relaxing activity of Topo I drives the elongation of transcription. Moreover, Topo I was shown to both activate and repress transcription in reconstituted transcription reactions [12], [13]. These effects did not require the relaxation activity of the protein and revealed a novel function of the enzyme in the regulation of transcription initiation by RNA Pol II. Recent data have shown that Topo I inhibition by camptothecin also activated promoter-proximal transcription, indicating a role for the enzyme in transcriptional pausing [14]. Therefore Topo I function in transcription is not limited to a simple unwinding of DNA during transcription elongation.

Topo I may participate in transcription through its action on SR proteins. These proteins constitute a conserved family of splicing factors that are required both for constitutive as well as alternative splicing, and are also involved in transport, translation and decay of mRNAs [3], [15]. More recently, SR proteins have also been implicated in transcription elongation and genomic stability [3]. Depletion of the SR protein SC35 diminishes the association of RNA Pol II with the kinase P-TEFb which phosphorylates the RNA Pol II C-Terminal Domain (CTD) and consequently dramatically reduces the production of nascent RNA [3], [16]. Through its capacity to phosphorylate SR proteins, Topo I has been shown to modulate SR protein activity in splicing [17], [18] and to prevent replication fork collapse by suppressing the formation of R-loops in an SR protein-dependent manner [19]. Therefore the presence of the topoisomerase and kinase activities in the same protein may fulfill functions required to coordinate transcription with RNA-processing events [4]. However, *in vivo* evidence implicating Topo I in RNA metabolism is lacking and this problem needs addressing with an integrated system.

In this study, we performed a genetic analysis in *Drosophila* to demonstrate that Topo I modulates the SR protein B52 phosphorylation status *in vivo*. In addition to perfect co-localization of both proteins on polytene chromosomes, we demonstrate that B52 targets Topo I to transcription sites. Impaired recruitment of Topo I following B52 depletion, induces an inefficient release of *hsp70* target mRNA from its transcription site and a delay in *hsp70* shutdown. These genetic findings raise the intriguing possibility that B52 and Topo I collaborate to release mRNPs and deactivate transcription of target genes and help to explain genomic instability and developmental defects associated with Topo I depletion in metazoa.

## Results

### *Drosophila* Topo I harbors an intrinsic kinase activity that modulates B52 phosphorylation *in vivo*

**Figure 1:**
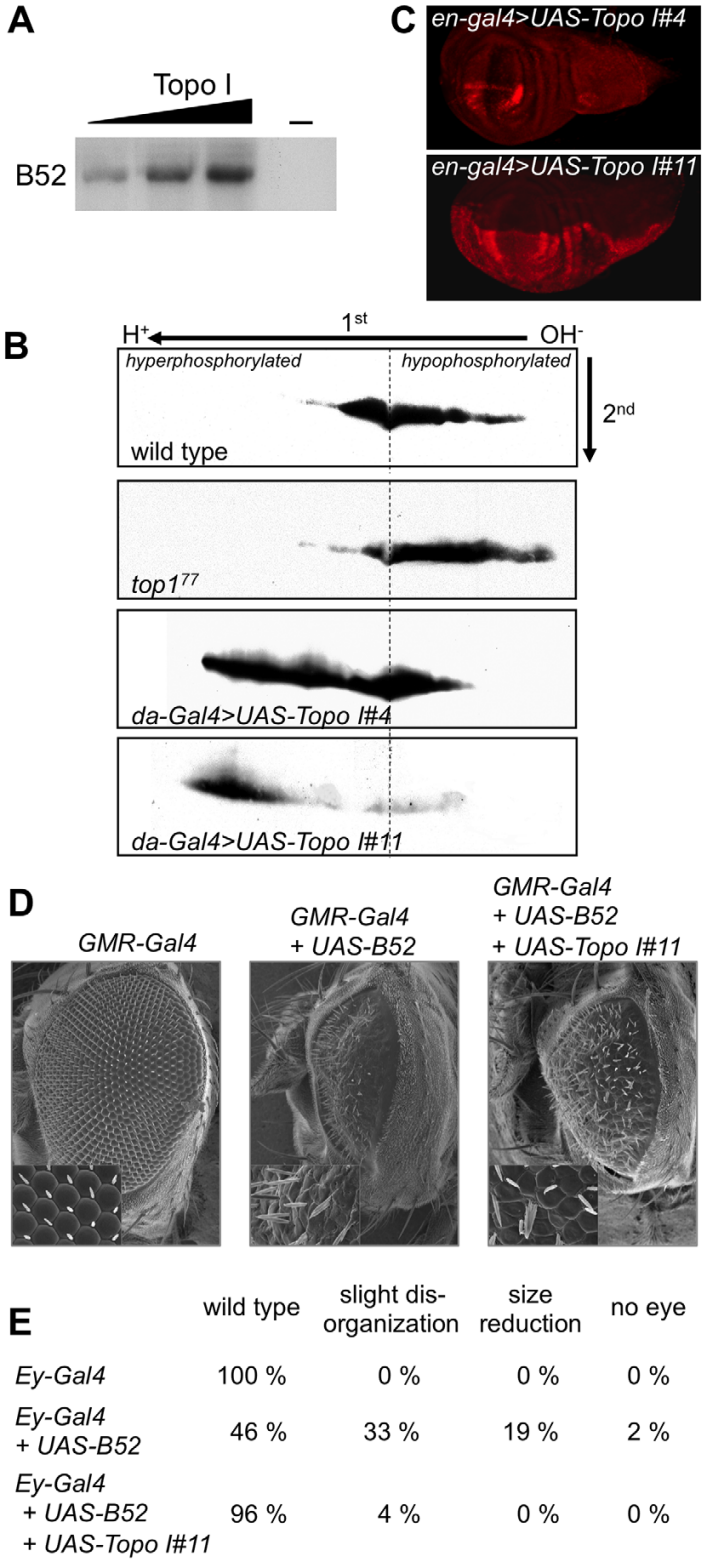
*Drosophila* Topo I phosphorylates B52 *in vitro* and *in vivo*. (A) *In vitro* analysis of Topo I kinase activity with purified Topo I and B52 in the presence of radioactive ATP. (B) Western blot analysis of B52 phosphorylation status after 2D electrophoresis, in wild type larvae (first panel), mutant *top1^77^* larvae (second panel), or in larvae ubiquitously overexpressing Topo I weakly (third panel, genotype: *UAS-Topo I#4/+, da-Gal4/+*) or strongly (last panel, genotype: *da-Gal4/UAS-Topo I#11*). (C) Immuno-staining of wing imaginal discs with anti-Topo I antibody, in *UAS-Topo I#4/+,en-Gal4/+* (up) and *en-Gal4/UAS-Topo I#11* larvae (bottom) showing that *UAS-Topo I#11* is more efficiently activated by GAL4 than *UAS-Topo I#4*. (D, E) *Topo I rescues the phenotypes induced by B52 overexpression*. Overexpression of B52 under the control of *GMR-Gal4* (D) or *ey-Gal4* (E) induces phenotypes in adult flies that are partially rescued by concomitant expression of Topo I. Phenotypes obtained with the *ey-Gal4* driver (E) are ranked according to their severity: wild-type, slight disorganization of ommatidia, reduction of eye size and total loss of eye. 100 to 150 eyes were scored.

Mammalian Topo I protein has an intrinsic kinase activity that phosphorylates the RS domain of SR proteins [20]. Kinase activity of Topo I has not yet been reported for other species. We first asked whether *Drosophila* Topo I can phosphorylate B52 protein *in vitro*. *Drosophila* Topo I was expressed and purified from SF9 cells, and incubated in the presence of radioactive ATP with purified B52 expressed in bacteria. Topo I phosphorylates B52 in a dose-dependant manner *in vitro* (Figure 1A), showing that the kinase activity of the protein is conserved in *Drosophila*. We then asked whether varying the level of Topo I *in vivo* could modify B52 phosphorylation status. To this end, proteins isolated from larvae were resolved on two-dimensional (2D) gels and B52 phosphorylation variants were analyzed by western blot. In wild type larvae, B52 migrates as a large population of spots revealing numerous post-translational modifications of the protein (Figure 1B). We first analyzed B52 phosphorylation in the Topo I loss-of-function mutant *top1^77^*, which is lethal at the second instar larval stage [8]. In *top1^77^* larvae, B52 is displaced towards the basic part of the gel (Figure 1B, panel *top1^77^*) indicating that B52 is less phosphorylated in this mutant. To test whether Topo I overexpression could increase B52 phosphorylation *in vivo*, we developed transgenic flies expressing the *top1* coding sequence under the control of *UAS* sequences (*UAS-Topo I*). Different insertions of the *UAS-Topo I* transgene displayed variable response to GAL4 due to position effects, as commonly observed. Figure 1C shows an example of this variation seen in the wing disc with the *en-Gal4* driver, which is expressed in the posterior part of each segment. In the *UAS-Topo I#4* line, a weak overexpression of Topo I was detected, whereas a strong overexpression was detected in the *UAS-Topo I#11* line. We expressed variable doses of Topo I under the control of the ubiquitous *da-Gal4* driver, using either the *UAS-Topo I#4* or the *UAS-Topo I#11* insertions. *da-Gal4/UAS-Topo I#4* individuals can survive to adulthood, whereas *da-Gal4/UAS-Topo I#11* individuals die as first or second instar larvae. This observation correlates with the strength of the transgenes expression. In *da-Gal4/UAS-Topo I#4* larvae, B52 distribution is extended towards the acidic part of the gel reflecting a high level of phosphorylation (Figure 1B, panel *da-Gal4>UAS-Topo I#4*). This effect is even more pronounced in *da-Gal4/UAS-Topo I#11* larvae, in which most of B52 is dramatically shifted towards the acidic part of the gel (Figure 1B, panel *da-Gal4>UAS-Topo I#11*). These results show that varying the Topo I level modulates B52 phosphorylation status *in vivo*; overexpression of Topo I increases B52 phosphorylation, whereas the Topo I loss-of-function mutant displays a hypo-phosphorylation of B52. Thus, *Drosophila* Topo I has a conserved kinase activity that modulates SR proteins phosphorylation *in vivo*.

To test if phosphorylation by Topo I regulated B52 activity, we asked whether Topo I overexpression could modify the phenotypes induced by B52 overexpression. We previously showed that B52 overexpression during eye development induces strong phenotypes in adult flies. Overexpression of B52 in the eye disc before differentiation, under the control of the *ey-Gal4* driver, gives rise to flies harboring variable loss of ommatidia with about 2% of flies totally lacking eyes [21]. Overexpression of B52 latter during ommatidia differentiation of the eye using the *GMR-Gal4* driver gives rise to strongly reduced and disorganized eyes [22]. We found that overexpression of Topo I significantly rescued the eye phenotypes induced by B52 overexpression with the *GMR-Gal4* driver (Figure 1D, panel *GMR-Gal4+UAS-B52+UAS-Topo I#11*) and the *ey-Gal4* driver (Figure 1E, *Ey-Gal4+UAS-B52+UAS-Topo I#11*). These results suggest that B52 hyper-phosphorylation mediated by Topo I represses its activity. Alternatively, given that B52 overexpression can alter Topo I localization (see below), the rescue can also be explained by a compensation of Topo I titration by B52.

### B52 and Topo I colocalize on polytene chromosomes

**Figure 2:**
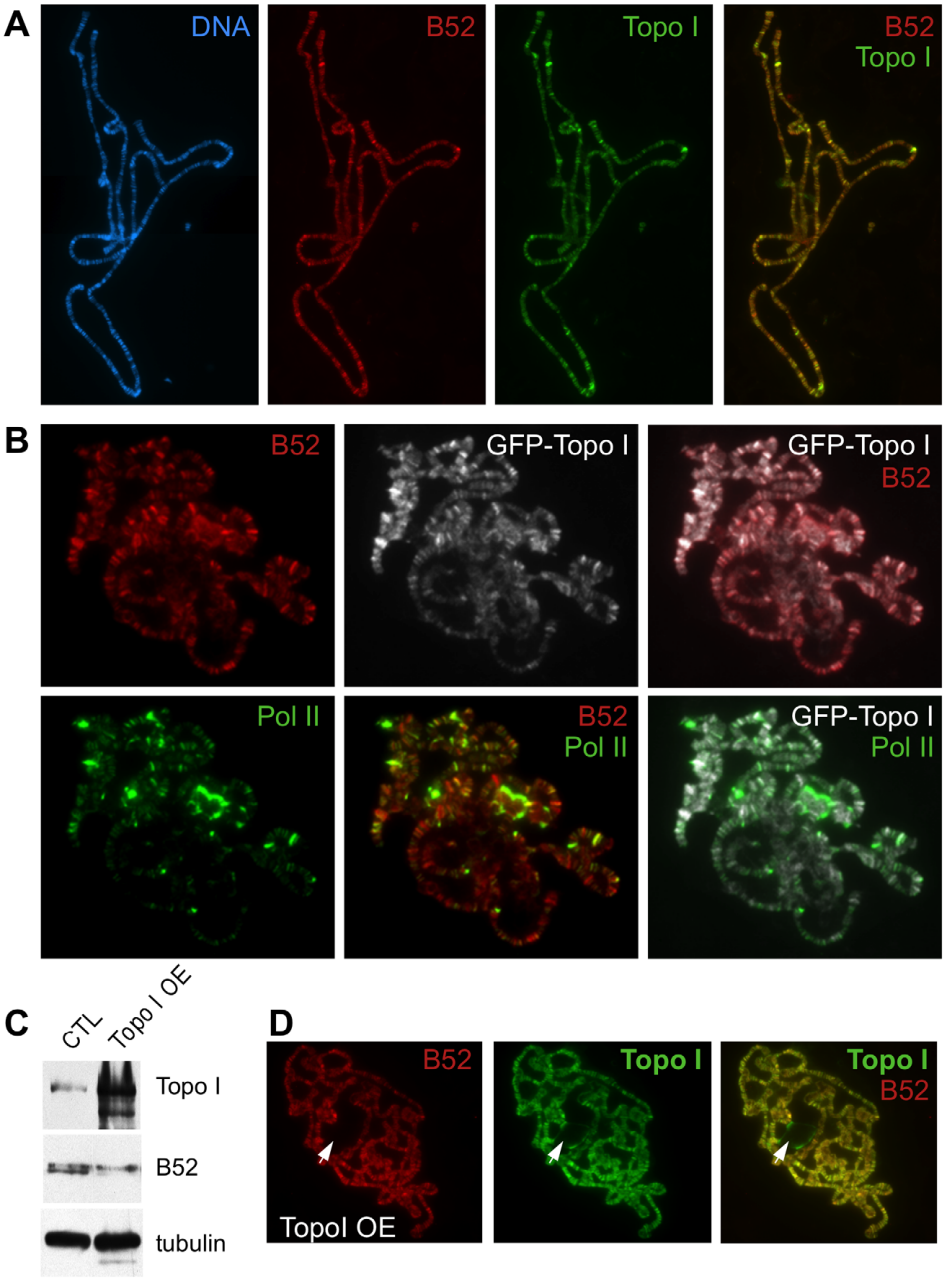
Analysis of B52 and Topo I protein distribution on polytene chromosomes squashes. (A) Chromosomes from wild type larvae stained with anti-B52 and anti-Topo I antibodies. The distribution of the bands, as well as their intensities, is almost identical. (B) Chromosomes from the *Wee-P153* line (expressing an endogenous GFP-Topo I fusion) stained with anti-B52, anti-GFP and anti-RNA Pol II Ser5P antibodies. The pattern of B52 and GFP staining is almost identical, as observed with B52 and Topo I antibodies. B52/Topo I distribution overlaps with RNA Pol II signal, nevertheless the intensities of the labeled bands do not always correlate. (C) Western blot analysis of control (genotype: *sgs3-Gal4*/+) or Topo I-overexpressing (Topo I OE, genotype: *sgs3-Gal4/UAS-Topo I#11*) salivary glands extracts. (D) Immunodetection of B52 and Topo I on polytene chromosome from salivary glands overexpressing Topo I (*sgs3-Gal4/UAS-Topo I#11* larvae). Note that B52 is not detected in the nucleolus (arrowhead) unlike Topo I.

Previous studies have separately analyzed the distribution of B52 and Topo I proteins on polytene chromosomes from salivary glands of third instar larvae. These proteins were shown to associate with transcriptionally active sites and to be rapidly recruited to heat shock genes following their activation by heat shock [23]–[25]. In order to map the relative localization of these proteins to each other and to RNA Pol II, we raised a rat serum against the last 16 amino acid residues of B52. We first compared the distribution of B52 and Topo I on wild type polytene chromosomes with the anti-B52 serum and anti-Topo I antibodies, which were detected by appropriate secondary antibodies. Remarkably, we observed an almost perfect overlap between B52 and Topo I staining patterns on the chromosome arms where most of the bands appeared as yellow signals reflecting colocalization of B52 (red) and Topo I (green) (Figure 2A). To ensure that there was no cross-hybridization with the secondary antibodies, we repeated the same experiment by omitting one of the primary antibodies, but keeping the two secondary antibodies. As expected, only one signal is detected with each primary antibodies, demonstrating absence of any cross detection (not shown).

To further address the colocalization of B52 and Topo I, we took advantage of the *Wee-P153* transgenic line that contains an insertion of a GFP cassette in the first intron of the *top1* gene [26]. This cassette is spliced into *top1* transcripts and results in an in-frame fusion of the GFP at the very N-terminus of Topo I, allowing detection of Topo I through the GFP. Consistent with colocalization of B52 and Topo I along chromosomes arms, a perfect overlay was observed between B52 and GFP signals in the *Wee-P153* line (Figure 2B). This distribution was then compared to that of RNA Pol II detected with the H14 monoclonal antibody, which recognizes the RNA Pol II CTD phosphorylated on Ser5. Consistent with previous reports, comparison of the RNA Pol II staining pattern (green) with either B52 (red) or Topo I (grey) staining revealed that not all chromosome sites have the same degree of staining (Figure 2B). Some sites were intensively stained with RNA Pol II, but only weakly with either B52 or Topo I (Figure 2B and S1). Conversely, staining of some B52 and Topo I sites did not always coincide with equivalent RNA Pol II staining (Figure S1). The biochemical basis for the differential distributions of Topo I/B52 proteins and RNA Pol II in some of chromosomal loci is unknown; however, the possibility that this was due to the nature of antibodies used can be ruled out, because similar results were obtained using different sets of anti-RNA Pol II and anti-Topo I antibodies [24], [25, this study]. Therefore, the observed variation in B52/Topo I *versus* RNA Pol II ratio probably reflects differential requirements for Topo I and B52 activity between transcribed loci.

### B52 chromosomal distribution is not affected by Topo I overexpression

Since Topo I overexpression can increase phosphorylation of B52 *in vivo*, we decided to analyze whether overexpression of Topo I in the salivary glands would perturb the localization of B52. To perform targeted overexpression of Topo I in the salivary gland, we drove expression of the *UAS-Topo I#11* transgene with the *sgs3-Gal4* line [27] that expresses GAL4 exclusively in this tissue from mid-third instar larval stage. Topo I overexpression in the salivary glands (Figure 2C, panel Topo I and Figure 2D) induced a shift of B52 mobility in western blots, reflecting hyper-phosphorylation of B52 (Figure 2C, panel B52). In this context, B52 (red) distribution was not significantly modified, as both proteins were still colocalized along the chromosomes arms (Figure 2D, merge Topo I and B52). Overexpression of Topo I enhances the banding pattern detected with the Topo I antibody, as several faint bands become very intense upon Topo I overexpression (Figure S2). Remarkably, these bands coincide with stronger B52 signals indicating that B52 recruitment to these sites is enhanced. Nevertheless, while specific accumulation of Topo I could readily be detected in the nucleolus, anti-B52 antibodies failed to stain this organelle (Figure 2D, arrowhead), implying that B52 does not accompany Topo I to the nucleolus.

### B52 overexpression titrates Topo I in the nucleoplasm

**Figure 3:**
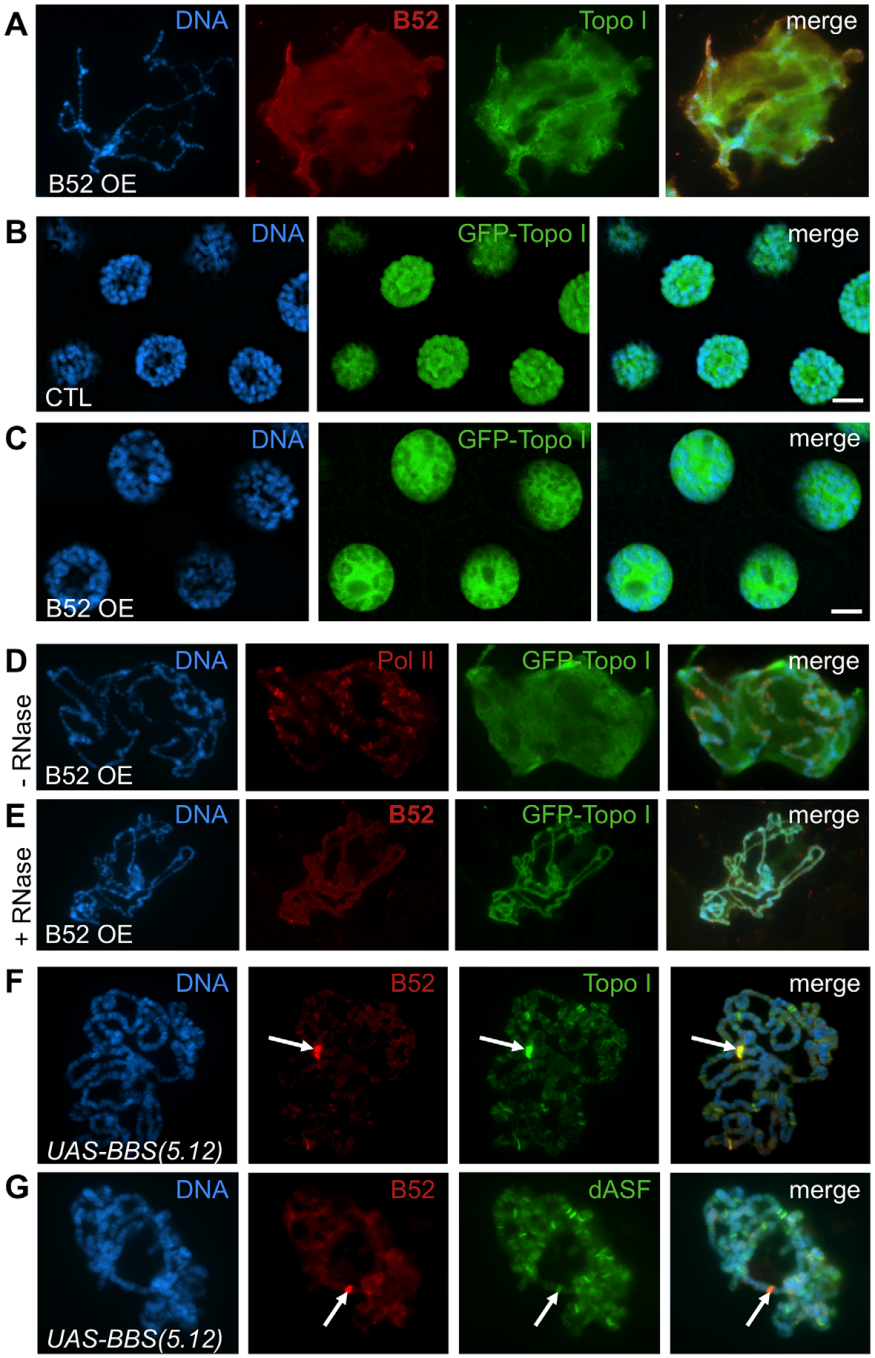
B52 influences Topo I localization. (A–E) *B52 overexpression induces nucleoplasmic accumulation of B52 and Topo I in an RNA-dependant manner*. (A) Squashed polytene chromosomes from B52-overexpressing (B52 OE) salivary glands, stained with anti-B52 and anti-Topo I antibodies. (B-C) Visualization of GFP-Topo I fluorescence in intact nuclei from *Wee-P153* line salivary glands in a wild-type (B) or in a B52-overexpressing context (C). (D–E) Immuno-staining of squashed polytenes chromosomes from the *Wee-P153* line over-expressing B52 in the salivary glands. In this context, GFP-Topo I accumulates in the nucleoplasm, whereas RNA Pol II is not affected. (E) RNase A treatment of the nuclei prior to fixation removes the nucleoplasmic staining of B52 and Topo I. (F-G) Immuno-staining of squashed polytenes chromosomes expressing the *UAS-BBS(5.12)* transgene, with anti-B52 and anti-Topo I antibodies (F) or with anti-B52 and anti-dASF antibodies (*G*). The position of the transgene, which titrates B52, is indicated by an arrow. Topo I is strongly recruited to this site compared to the SR protein dASF. Genotypes: (A) *w/Y; sgs3-Gal4/UAS-B52*. (B) *y,w,Wee-P153/Y; sgs3-Gal4/+*. (C-E) *y,w,Wee-P153/Y; sgs3-Gal4/UAS-B52.* (F,G) *sgs3-Gal4/UAS-BBS(5.12)*.

Given that Topo I suppresses phenotypes induced by B52 overexpression (Figure 1D, E), it was important to determine the consequences of B52 overexpression on Topo I localization. B52 overexpression in the salivary glands dramatically altered Topo I distribution. Both B52 (red) and Topo I (green) accumulated around the squashed chromosomes (Figure 3A). We confirmed this observation in the *WeeP-153* line: in B52-overexpressing nuclei, GFP-Topo I accumulated around the squashed chromosomes as did B52 (Figure S3). To ascertain that the relocalization of Topo I seen on chromosomes spreads is not due to a technical artifact, we analyzed the distribution of GFP-Topo I in intact salivary gland nuclei, where GFP fluorescence is preserved compared to the squashed preparation of chromosomes. In wild type nuclei, GFP-Topo I is present on the chromosome arms, and in the nucleolus (Figure 3B). Overexpression of B52 in the salivary glands of the *Wee-P153* line induces a massive relocalization of GFP-Topo I from the chromosomes and the nucleolus, into the nucleoplasm (Figure 3C). Interestingly, we observed that B52 overexpression in the salivary gland nuclei led to an increase of the nuclear size (Figure 3, compare B and C).

Whereas Topo I is redistributed in the nucleoplasm, RNA Pol II distribution was not affected by B52 overexpression (Figure 3D, panel Pol II in red) indicating that B52 does not displace transcription factor complexes, but more specifically affects Topo I recruitment on chromatin. In order to determine whether RNA could participate in the accumulation of B52 and Topo I in the nucleoplasm of B52-overexpressing nuclei, salivary glands were incubated in the presence of RNase A prior to fixation and squashed chromosomes were analyzed as before. RNase A treatment abolished the nucleoplasmic signal seen in B52-overexpressing nuclei (Figure 3E), indicating that nucleoplasmic accumulation of B52 and Topo I is RNA-dependant.

### B52 recruits Topo I to transcription sites

Physical interaction between Topo I and SR proteins has been shown to modulate both DNA relaxation and SR protein phosphorylating activities in mammals [28]–[31]. To determine whether the *Drosophila* SR protein B52 can recruit Topo I by direct interaction, we made use of the *UAS-BBS(5.12)* transgene that expresses an inhibitory aptamer RNA (iaRNA) containing stretches of high-affinity B52-Binding Sites (BBS) [32]. Expression of this iaRNA under the control of a heat shock promoter was previously shown to recruit B52 to the transgene transcription site on polytene chromosomes after induction by heat shock [32]. In our assay, targeted expression of *UAS-BBS(5.12)* in the salivary gland under the control of the *sgs3-Gal4* driver, led to a titration of B52 at the transgene site (confirmed by immuno-FISH experiments, data not shown) with a concomitant decrease of B52 signal along the chromosomes (Figure 3F, panel B52 in red). In this context of B52 titration with BBS, massive recruitment of Topo I was similarly observed at the transgene insertion site (Figure 3F, panel Topo I in green). The same result was observed with the GFP-tagged Topo I when the *UAS-BBS(5.12)* transgene was expressed in the *Wee-P153* line: both B52 and GFP-Topo I are strongly recruited to the transgene upon activation of its expression by GAL4 (Figure S4). As a control, we analyzed the distribution of the SR protein dASF in the same context as for B52. Unlike B52, dASF was not recruited to the *UAS-BBS(5.12)* transcription site (Figure 3G, panel dASF in green).

**Figure 4:**
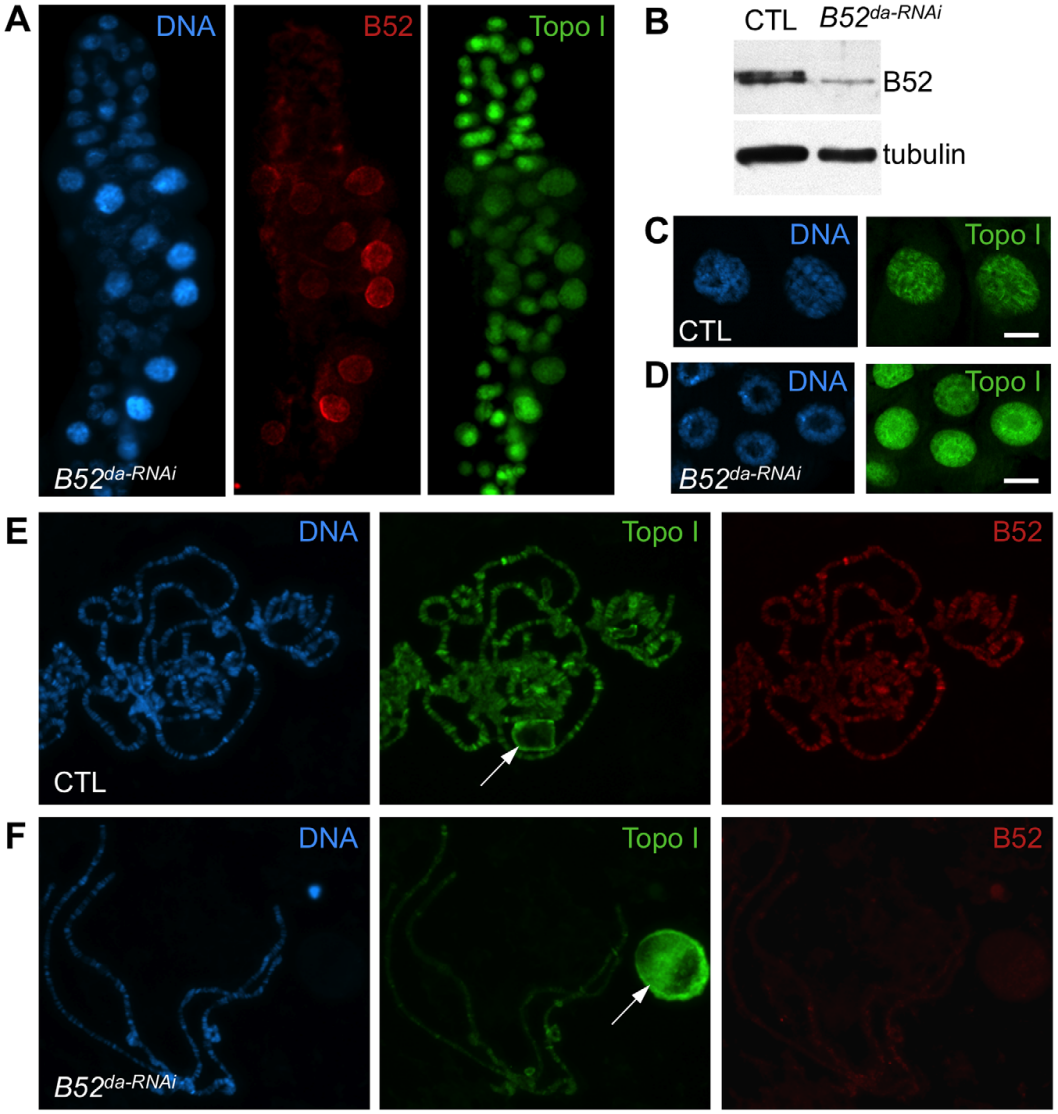
B52 depletion induces accumulation of Topo I in the nucleolus. (A) Immunostaining of salivary gland from larvae expressing inverted repeats against B52 (*B52* ^da-RNAi^, genotype: *da-Gal4/UAS-IR-B52*). Several nuclei escape the RNAi effect and show B52 labeling and diffuse Topo I localization, whereas B52-depleted cells, which are smaller, display a bright spot of Topo I staining in the center of the nuclei. (B) Immunoblot on salivary gland extracts from control (*da-Gal4*/+) or B52-depleted (*da-Gal4/UAS-IR-B52*) larvae. (C,D) Optical section through the middle of salivary glands nuclei from control (C) and *B52* ^da-RNAi^ (*D*) third instar larvae, stained with anti-Topo I antibody. (E,F) Immuno-staining of squashed polytenes chromosomes from control (*da-Gal4/+*) and *B52^da-RNAi^* (F) third instar larvae salivary glands.

To further analyze the implication of B52 in the process of Topo I recruitment to transcription sites, Topo I distribution was tested in the context of B52 depletion. Since B52 loss-of-function mutants are lethal at the second instar larval stage, we attempted to deplete B52 by RNAi to obtain third instar larvae from which polytene chromosomes preparation is more convenient. To this end, we used a *UAS-IR-B52* transgene which carries an inverted repeat hairpin targeting B52 under the control of *UAS* sequences. Expression of the *UAS-IR-B52* transgene under the control of the ubiquitous *da-Gal4* driver (thereafter called *B52^da-RNAi^*) induced lethality at 25°C, mainly during the pupal stage, with only rare adults recovered. Notably, *B52^da-RNAi^* third instar larvae displayed smaller salivary glands and western blot analysis showed that B52 was significantly depleted and hypo-phosphorylated in *B52^da-RNAi^* salivary glands (Figure 4B, panel B52). Immunostaining of these salivary glands revealed that B52 depletion is not always efficient in all cells, and that B52-depleted cells are smaller than B52-positive cells (Figure 4A, panel B52 in red). This peculiar property offers the opportunity to compare B52-depleted cells to control cells in the same salivary gland. We observed that Topo I distribution was dramatically different between wild type and B52-depleted nuclei; whereas Topo I is mainly present on the chromosome arms in wild type cells (Figure 4C), most Topo I staining was detected in the nucleolus of B52 depleted nuclei (Figure 4D). The accumulation of Topo I in the nucleolus was confirmed by co-staining with anti-fibrillarin antibodies (Figure S5). This effect was strongly evident on squashed preparations of polytene chromosomes. To allow a robust comparison of immuno-stainings, wild type and *B52^da-RNAi^* salivary glands were mixed on the same slide and treated as unique sample. Chromosomes from *B52^da-RNAi^* larvae, which are clearly identified by the absence of B52 signal, displayed a faint Topo I signal on the chromosome arms, with fewer bands compared to wild type (Figure 4E and F compare panels Topo I in green). Topo I signal was, however, mainly detected in enlarged nucleoli (Figure 4F). These results indicate that B52 depletion impairs localization of Topo I on polytene chromosomes, allowing its accumulation in the nucleolus.

### Depletion of B52 impairs *hsp70* shutdown and *hsp70* mRNA release

**Figure 5:**
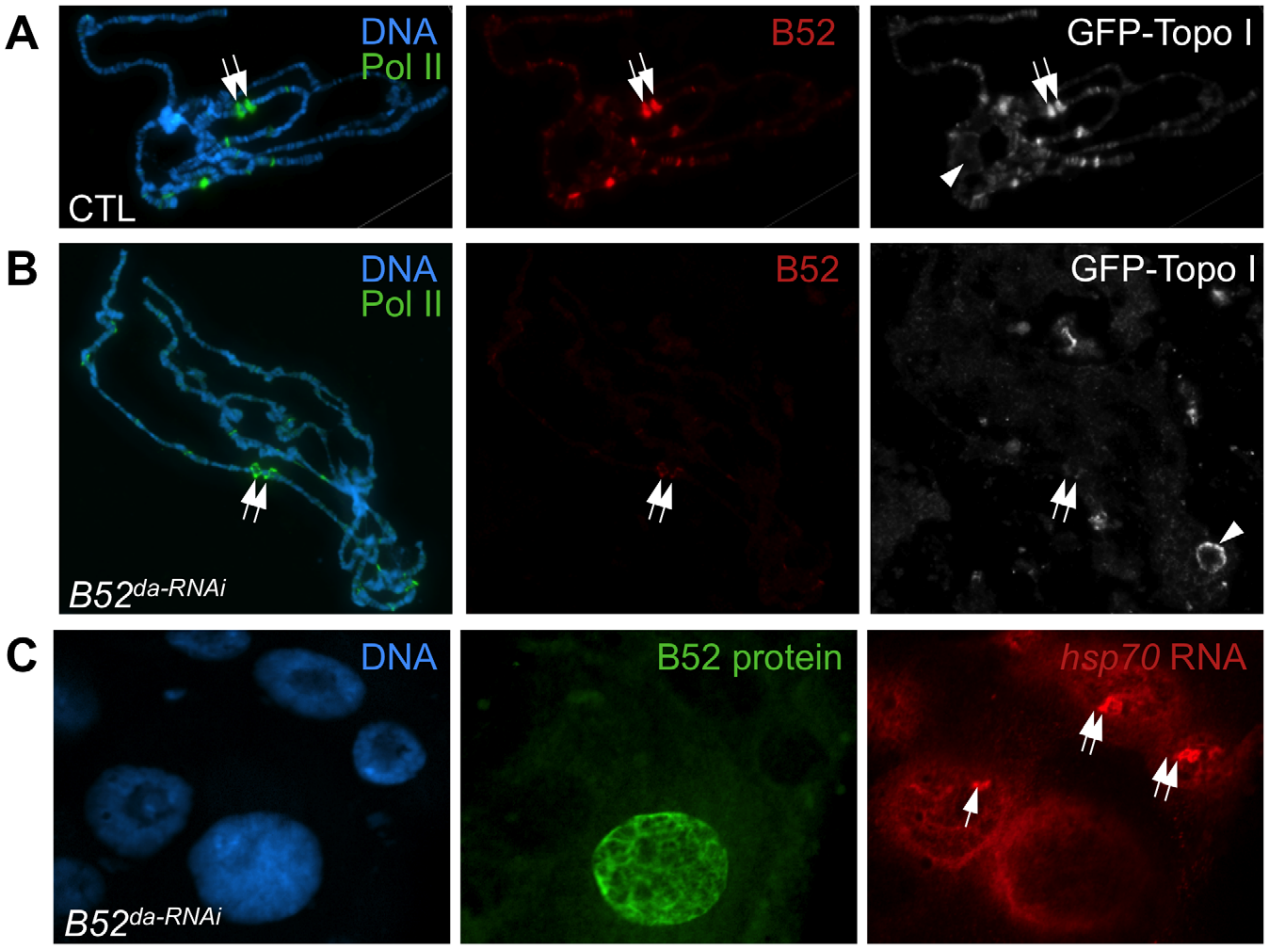
B52 depletion impairs Topo I recruitment at the *HS* gene transcription sites and *hsp70* mRNA release. (A-B) Immuno-staining of squashed polytenes chromosomes from control (*da-Gal4/+*) and *B52^da-RNAi^* larvae after heat shock, with anti-RNA Pol II ser5P, anti-B52 and anti-Topo I. The positions of the *hsp70* loci are indicated by arrows. (C) Analysis of *hsp70* RNA localization by *in situ* hybridization in B52-depleted (*da-Gal4/UAS-IR-B52*) salivary glands, after 20 min heat shock, combined with immuno-detection of B52 protein. *hsp70* transcription sites are indicated by arrows. In this experiment, one cell escapes the RNAi effect and still expresses B52. In this cell *hsp70* RNA does not accumulate at its transcription site.

Both B52 and Topo I were previously shown to be strongly recruited to *heat shock* (*HS*) genes after their induction by heat shock [23]–[25]. As most *HS* genes lack introns, this observation suggests a splicing-independent role of B52 at these sites. Based on above observations, B52 could also participate to Topo I recruitment on *HS* genes. Therefore, we analyzed Topo I and RNA Pol II recruitment on *HS* loci after induction by heat shock in B52-depleted polytene chromosomes, compared to the wild type. We used the endogenous GFP-tagged Topo I for convenience. As expected, both GFP-Topo I (grey) and B52 (red) are recruited to *HS* genes after heat shock induction (Figure 5A). However, GFP-Topo I recruitment on *HS* loci is dramatically reduced in B52-depleted chromosomes (Figure 5B), whereas RNA Pol II (green) is still detected after heat shock (compare Figure 5A and B). It should be noted that B52 depletion by RNAi is not complete. Low levels of B52, which is barely detectable on chromosomes in the absence of heat shock (Figure 4F), become concentrated on *HS* loci after activation. This residual B52 could be the cause of moderate detection of Topo I at *HS* loci. Altogether, these data show that B52 plays a major role in Topo I recruitment to *HS* transcription sites.

**Figure 6:**
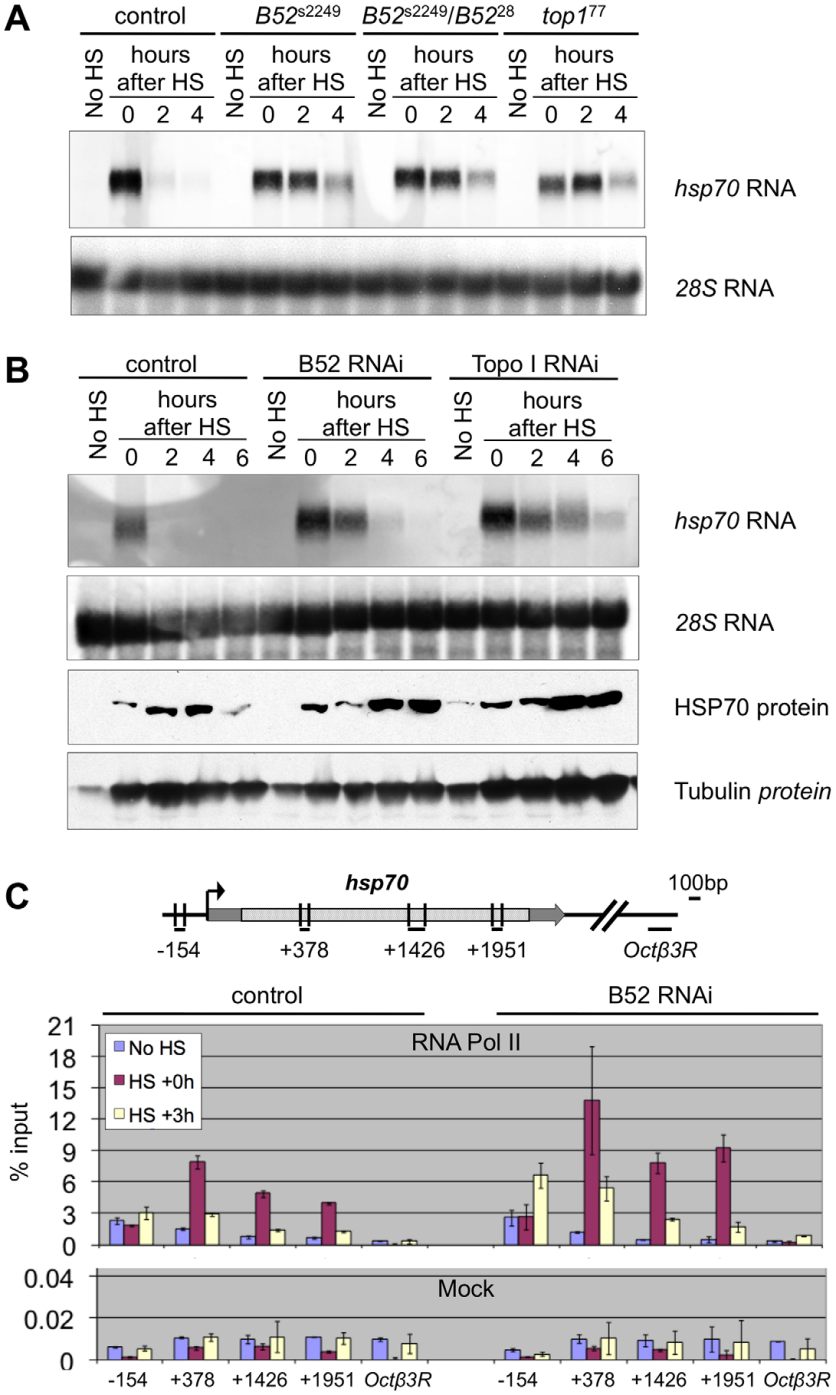
B52 and Topo I are required for *hsp70* transcription shutdown. (A) Kinetic analysis of *hsp70* mRNA expression by northern blot before (No HS) and after 40 min heat induction in control (*w* ^1118^), *B52* mutants (*B52* ^s2249^ homozygotes and *B52* ^28^/*B52* ^s2249^ heterozygotes), and *Top1* mutant (*top1* ^77^) larvae. 28S rRNA serves as a loading control. (B) Kinetic analysis of *hsp70* expression before (No HS) and after 40 min heat induction in control by northern blot and western blot on control (*da-Gal4*/+), B52-depleted (*da-Gal4/UAS-IR-B52*) and Topo I-depleted (*da-Gal4/UAS-IR-Top1*) third instar larvae. (C) Distribution of RNA Pol II on *hsp70* gene determined by Chromatin-ImmunoPrecipitation and quantitative PCR. The primer pairs are indicated below the *hsp70* gene model. The histogram represents the percentage of chromatin immunoprecipitated by anti-RNA Pol II antibody (Top panel) or no antibody (Mock, bottom panel), before heat shock (no HS), just after 40 min heat activation (HS+0) or after 3 h of recovery following HS (HS+3 h).

To determine the functional consequences of the B52-mediated recruitment of Topo I to *HS* genes transcription, we focused our study on the intron-less *hsp70* gene which enabled us to analyze B52 function independently of its role in RNA splicing. We first performed a kinetic analysis of *hsp70* mRNA expression by northern blot in wild type, B52 mutants and Topo I mutant larvae (Figure 6A). In the wild type situation, *hsp70* mRNA was strongly detected immediately after 40 min heat shock (Figure 6A, Control, lane 0), and disappeared almost completely 2 hours after heat shock (see lanes 2 and 4 hours after HS), due to short mRNA half-life and to *hsp70* transcription shut down [33]. To analyze *hsp70* expression in B52 and Topo I mutant backgrounds, we used both the available mutants or RNAi-mediated depletions. Since the original *B52* mutant, *B52* ^28^, contains a deletion that disrupts both *B52* and the neighboring gene *Hrb87F*, we used this mutant in combination with the *B52* ^s2249^ allele which contains an insertion of a transposable element in the 5′UTR of the *B52* gene. This mutant dies at the second and third instar larval stages. As in the wild type, *hsp70* mRNA was strongly induced after heat shock in *B52* ^28^/*B52* ^s2249^ heterozygote as well as in *B52* ^s2249^ homozygote larvae (Figure 6A). This result is consistent with previous data showing that induction of *hsp70* is not impaired in the *B52* ^28^ mutant [34]. However, in this genetic background *hsp70* mRNA is still detected 4 hours after the end of the heat shock. The same result was obtained in *B52* ^da-RNAi^ larvae that express an RNAi against *B52* (Figure 6B). This observation may reflect changes in *hsp70* mRNA decay or a prolonged expression of the gene after the end of the heat shock. Strikingly, the same results were obtained in the *top1* ^77^ mutant (Figure 6A) as well as in larvae expressing an RNAi against Topo I (*top1* ^da-RNAi^) (Figure 6B). While *hsp70* induction is not impaired by Topo I depletion, *hsp70* mRNA is still detected 4 to 6 hours after the end of the heat shock.

These results are reminiscent of the defects observed in *Drosophila* mutants for the P68 RNA helicase. In *p68* mutants, *hsp70* mRNA gene deactivation is delayed, and this correlates with impaired release of *hsp70* mRNA from its transcription sites [35]. Therefore, we asked whether B52 depletion could also impair *hsp70* mRNA release. We analyzed *hsp70* mRNA distribution in salivary glands from *B52^da-RNAi^* larvae by *in situ* hybridization (Figure 5C). After 20 min heat shock, *hsp70* mRNA (red) strongly accumulates at its transcription sites in B52-depleted cells, but never in B52-positive cells (Figure 5C). Despite its accumulation at transcription sites, *hsp70* mRNA is clearly detected in the nucleoplasm of B52-depleted nuclei, indicating that *hsp70* mRNA export is not completely blocked. Consistently, immunoblot analyses showed that HSP70 protein is expressed after heat shock in B52- and Topo I-depleted larvae (Figure 6B), indicating that *hsp70* mRNA is properly processed, exported and translated in these mutants. Remarkably, HSP70 protein level continues to increase 4 to 6 hours after the end of the HS in B52- and Topo I-depleted larvae compared to wild type. This is consistent with the prolonged expression of the *hsp70* mRNA detected by northern blot. Taken together, our results show that B52 depletion impairs *hsp70* mRNA release from its transcription sites and causes an extended expression of *hsp70* after HS, suggesting a defect in transcription deactivation.

To directly address this hypothesis, we analyzed the distribution of RNA Pol II on the *hsp70* gene by chromatin immunoprecipitation after cross-link (X-ChIP) with anti-RNA Pol II antibodies, followed by quantitative PCR (q-PCR). We reasoned that if transcription shutdown is impaired by B52 depletion, we should be able to detect RNA Pol II on the *hsp70* genes several hours after HS. We analyzed four regions covering the *hsp70* gene and a negative control, in wild type and B52-depleted larvae, at 3 time points: before HS, just after HS and 3 h after the end of the HS (Figure 6C). As expected, we observe that RNA Pol II is strongly recruited on *hsp70* after HS, both in WT and B52-depleted contexts. Remarkably, 3 h after the end of the HS, we detect more RNA Pol II on *hsp70* in the B52-depleted context (roughly 2 fold) than in the wild type. This result provides a direct evidence that *hsp70* transcription is maintained at a higher level in B52-depleted larvae than in wild type. Altogether our results show that B52 is required for efficient *hsp70* mRNA release from its site of transcription and shutdown of *hsp70* transcription after heat shock.

## Discussion

SR protein splicing factors can interact with mammalian Topo I as a recombinant protein *in vitro* and they coimmunoprecipitate from nuclear extract [29]–[31]. This interaction between Topo I and SR proteins allows Topo I to achieve specific phosphorylation of SR proteins [29], [30], [36]. Thus, depletion of Topo I from mammalian cells results in the hypo-phosphorylation of SR proteins, impaired SR protein-dependent splicing and increased genomic instability due to R-loop formation [18], [19]. The present study provides compelling *in vivo* evidence that the targeting of Topo I to active transcription sites is part of the mechanism by which the SR protein B52 functions in *Drosophila*. B52 was only previously known to be important for regulating alternative splicing [34], [37], [38]. However, this mechanism cannot explain the recruitment of B52 to intron-less genes like heat shock (*HS)* genes [23], [24]. The delivery of Topo I by B52 may represent a novel mechanism involved in the control of gene expression. In *Drosophila* salivary glands both B52 and Topo I are strongly recruited to *HS* genes after heat shock ([23], [24] and here Figure 5) but depletion of neither protein affected *hsp70* gene induction ([34], [37] and Figure 6A,B). B52-mediated recruitment of Topo I to the activated *hsp70* loci appears to be essential for efficient release of *hsp70* mRNA from its transcription sites and to turn off transcription. In B52- or Topo I-depleted cells, expression of *hsp70* is abnormally maintained several hours after the end of induction (Figure 6). Also, *hsp70* mRNA is strongly retained at its transcription sites when B52 is depleted (Figure 5C). These findings support the notion that efficient release of mRNPs and transcription shutdown are coupled processes requiring targeted recruitment of Topo I to active chromatin by B52.

Because of its ability to dissolve both positive and negative supercoils from constrained DNA, it has been proposed that Topo I provides swivels for removing torsional constraint that accompanies DNA-associated processes [14]. However, there is no evidence that Topo I alone directly drives the elongation of transcription through its relaxing activity. In fact, the DNA relaxation activity has been shown to be dispensable for both repression and activation of transcription in reconstituted transcription reactions [12], [13]. Studies with yeast DNA topoisomerase mutants further support the idea that Topo I is not essential for transcription by RNA Pol II [7], [11], [39]. However, plasmids carrying transcriptionally active genes are found to be extremely negatively supercoiled when isolated from mutants lacking Topo I [39], implying that a major function of Topo I is to remove negative supercoils after transcription elongation. Moreover, Topo I mutants including strains with null mutations are viable and exhibit no obvious growth defects, demonstrating that Topo I is not essential for viability in yeast [7], [11], [39]. Rather, yeast Topo I seems to be required for gene repression during a critical period when cells approach and enter the stationary phase [40]. In contrast, depletion of zygotic and maternal Topo I in *Drosophila* leads to an early embryonic lethal phenotype [41]. The data presented here indicate that Topo I is involved in programmed gene shut-off at *hsp70* locus, which likely plays a complementary and essential role to gene activation during *Drosophila* development. The mechanisms that are responsible for transcription attenuation during the recovery phase after heat shock are not precisely known. Studies in many eukaryotes have established that *heat shock* genes repression requires the heat shock protein themselves, which participate in a negative autoregulation loop. In mammals for example, HSP70 protein associates with HSF1 and represses its transcriptional activity, thereby repressing *heat shock* genes transcription [37], [42]. Moreover, it was shown in *Drosophila* that a certain level of HSP70 protein produced is necessary to trigger repression of *HS* genes transcription [33], [37]. In our experiments, we observed that HSP70 protein is expressed but continues to accumulate several hours after HS in the B52-depleted context, compared to the wild type. Therefore the amount of HSP70 protein is certainly not limiting for the autoregulation to take place. Our results more likely suggest that B52 and Topo I participate to *hsp70* transcription shutdown by promoting *hsp70* mRNA clearance from its transcription sites, as it has been shown for the RNA helicase P68 [35], [37]. Gene activation involves both changes in chromatin structure and assembly of maturing transcripts with complexes involved in their initiation, elongation, termination and processing [1]. Because of such intimate coupling, many RNA-binding proteins are found in close contact with template DNA, which is detectable by chromatin immunoprecipitation (ChIP) in all eukaryotic cells [43], [44]. Among these factors are members of the SR protein family. Thus, to coordinate mRNP release and transcription shutdown Topo I has acquired novel properties. Among these, are the abilities of Topo I to phosphorylate SR proteins [4], [20] and to interact with RNA-binding proteins involved in pre-mRNA splicing like SF2/ASF, hnRNP A/B, PSF and p54^nrb^ [17], [29]–[31].

Recently, a proteomic analysis of Topo I-purified complexes identified 36 Topo I partners, 24 of which are involved in RNA metabolism [45]. These include nucleolin, a nucleolar phosphoprotein, which is implicated in the synthesis and maturation of ribosomes [46]. Consistent with the requirement for Topo I during ribosomal RNA synthesis, Topo I interacts also with RNA polymerase I [11], [39], [47]. Furthermore, the most prominent phenotype associated with *top1* mutants in yeast is an altered chromatin structure at the rDNA locus [11], [39], [47], [48], suggesting that the enzyme might be involved in chromatin remodeling at this locus. Given that B52 depletion in *Drosophila* leads to nucleolar accumulation of Topo I (Figure 4F), it is possible that proteins with RRM domain, like nucleolin, are involved in the localization of Topo I in the nucleolus. SR proteins could compete with nucleolar protein(s) for the distribution of Topo I between the nucleolus and nucleoplasm (Figures 3 and 4). These data invoke a striking parallel between coordination of transcription and processing events for RNA Pol II and RNA Pol I by SR proteins and nucleolin respectively. Similar to SR proteins function at RNA Pol II loci, the interaction of nucleolin with nascent pre-rRNA has been shown to help the co-transcriptional assembly on pre-rRNA of factors necessary for the subsequent maturation of the pre-ribosomal particle [46]. Thus, the involvement of nucleolin at multiple steps of ribosomal RNA biogenesis pathway suggests that it could play a key role in this highly integrated process including the release of rRNP particles. It is not known whether the kinase activity of Topo I is also required in the nucleolus. Interestingly, the interaction between the RNA-binding domains of the SR protein SF2/ASF and Topo I inhibits its DNA relaxation activity without affecting its kinase activity [29] therefore it is tempting to speculate that the RNA-binding domain of nucleolin and/or other nucleolar RNA-binding proteins could similarly inhibit the relaxing activity of Topo I and/or act as a mediator of Topo I localization.

**Figure 7:**
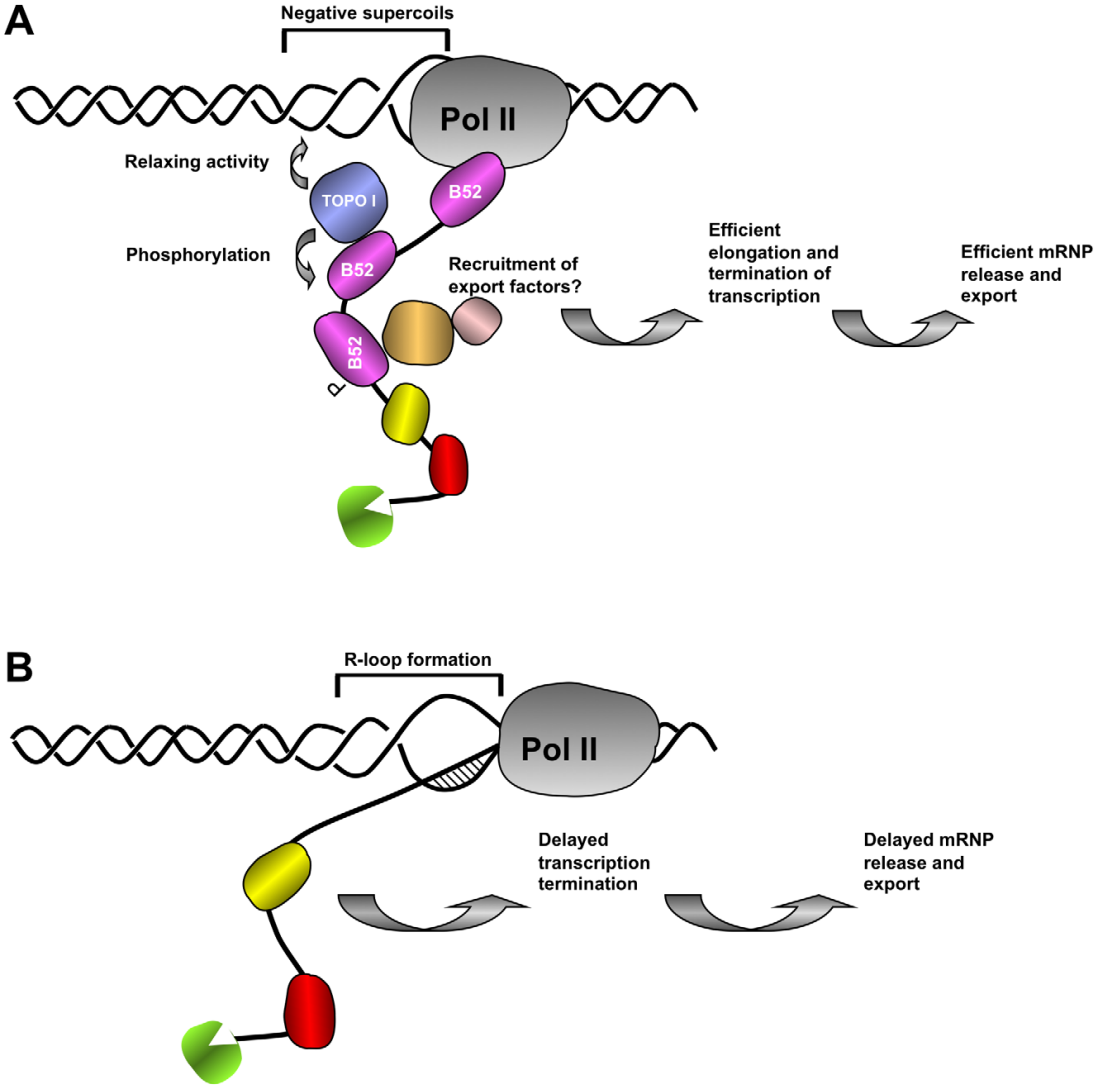
Proposed model of the coordination between nascent mRNP release and DNA supercoiling mediated by SR protein B52 and Topo I. (A) In wild type nuclei, B52 (purple) and other RNA binding proteins (green, yellow and red) travel with transcribing polymerase to scan emerging transcript for cis acting sequences and help release of RNA from the DNA template. B52 recruits Topo I both to remove negative supercoils generated behind the polymerase, preventing formation of large extended single stranded DNA and to mediate its phosphorylation, thereby changing its interaction properties with other partners of the export machinery. (B) In the absence of B52, recruitment of Topo I is impaired leading to R-loop formation between nascent transcript and negatively supercoiled DNA template thereby retarding elongation of RNA Pol II complex and release of export-competent mRNP necessary for transcription shutdown.

Impaired removal of nascent RNA from transcription sites leads to formation of RNA/DNA hybrids known as R-loops that trigger genomic instability [2], [19], [49]–[51]. Loss of mammalian SF2/ASF has been shown to lead to extensive pairing between the nascent transcripts and template DNA, allowing R-loop formation and consequently genomic instability [52] and Topo I is the major activity that suppresses the deleterious effect of R-loop formation in *E. coli* [50] and in mammals [19]. In *E. coli*, transcribed RNA is translated directly, whereas in mammals primary transcripts are processed and associated with export factors in the nucleus before being translated in the cytoplasm. So translation and processing have evolved to protect against R-loops in bacteria and eukaryotes, respectively. R-loop formation is more extensive on hypernegatively supercoiled templates that are generated following Topo I depletion in different organisms [2], [19], [50]. Since transcription-induced R-loop formation is a problem in all organisms [52], B52-mediated loading of Topo I on RNPs may have evolved to avoid R-loop formation in *Drosophila*. One function of B52 is to help to restore unconstrained DNA conformation by bringing Topo I to sites of active transcription (see model Figure 7). On the other hand, B52 facilitates the release of mRNAs and their export to keep transcripts away from actively transcribed DNA and thereby prevent its re-hybridization with DNA from which it originates.

Works in a variety of organisms suggest that newly assembled mRNPs are tethered to chromatin to ensure that only properly processed RNAs are released for export [2]. Depletion of factors, including B52, that are involved in RNP processing and export leads to mRNP accumulation at the site of transcription (Figure 5C). In mammals SR proteins have been shown to interact with the export factors TAP(NXF1)/p15 and facilitate export [53]. Studies of RNA export in *Drosophila* suggest that the pathway has been highly conserved [54]. Interaction between B52 and *Drosophila* NXF1/P15 may allow nascent transcripts to leave their sites of synthesis and undergo transport to nuclear pores. Since this interaction is dependent on the phosphorylation status of SR proteins in mammals [53], it can be assumed that Topo I-mediated phosphorylation could similarly have an impact on mRNP release and export (see model presented Figure 7).

Recently, we have shown that mammalian Topo I deficient cells, unlike yeast *top1*Δ mutants, display an increased rate of fork stalling during DNA replication due to R-loop formation [19]. Absence of a kinase activity associated with yeast Topo I could explain the difference in the phenotype between mammalian and yeast Topo I-deficient cells [19]. Consistent with our model that Topo I/kinase as well as SR proteins interfere with R-loop formation, is the observation that inhibition of this activity with Diospyrin [55] or depletion of ASF/SF2 in cells expressing Topo I [19] reproduces the replication defects and genomic instability of Topo I-deficient cells. These defects are, however, fully suppressed by inhibiting transcription or by degrading DNA-RNA hybrids with RNase H [19]. Thus, SR protein-mediated Topo I recruitment to active transcription sites and subsequent transcription shutdown is likely to be a key mechanism for preventing genomic instability during DNA replication. Consistent with this prediction is the finding that chromosome breaks preferentially occur at gene-rich regions in the absence of Topo I and their accumulation was directly proportional to the level of gene expression [19]. Interestingly, chromosomal breaks in Topo I-deficient cells accumulate at replication-dependent histone genes which are highly expressed in S-phase and are rapidly downregulated when DNA replication is repressed [56], [57]. Failure to shutdown histone genes expression could be a major source of interference between replication and transcription and could explain differences in the sensitivity of various cell types to specific inhibitors of Topo I, like CPT and its derivatives [5], [58].

## Materials and Methods

### Fly strains, transgenic lines and *Drosophila* genetics

The B52 mutant *B52* ^28^, as well as *UAS-B52* and *UAS-BBS(5.12)* transgenic lines were kindly provided by John Lis. The mutants *B52* ^s2249^ and *top1* ^77^ were obtained from Bloomington *Drosophila* stock center. *B52* ^28^ contains a deletion that disrupts both *B52* and the neighboring gene *Hrb87F* [37]. *B52* ^s2249^ corresponds to an insertion of a transposable element in the 5′UTR of the *B52* gene (Flybase). *top1* ^77^ contains a rearrangement in the 3′ region of the *top1* gene [8]. These mutants die at the second instar larval stage. The *Wee-P153* line [26] was kindly provided by Graeme Davis. The *UAS-IR-B52* and *UAS-IR-top1* transgenic lines were obtained from the Vienna *Drosophila* RNAi Center. *UAS-Topo I* transgenic flies were generated as follows. The *Drosophila* Topo I open reading frame (ORF) was PCR-amplified from a clone containing a *top1* cDNA (pcTop1-2 clone, kindly provided by Tao Hsieh) and cloned in the *Bgl*II and *Xba*I sites of *pUAST*. This construct was injected into *w* ^1118^ embryos together with a source of *P* transposase using conventional methods.

### Kinase assays and 2D gels

A *Bgl*II-*Xba*I PCR fragment corresponding to the *Drosophila* Topo I coding sequence was cloned in the *BamH*I and *Xba*I sites of *pFastBac1* expression vector (Invitrogen). Topo I protein was expressed in SF9 cells and purified on nickel columns *via* an endogenous stretch of 7 histidines present at the very N-terminus of the protein. B52 was expressed in bacteria as a His-tagged protein and purified on NiNTA agarose beads (Qiagen). Kinase assays were performed as described in [30]. 2D gels were performed as described in [17]. B52 was detected by the mAb104 antibody followed by ECL staining.

### Immuno-stainings

Immuno-staining of polytene chromosomes was performed as described in [59]. Larvae were raised at 25°C instead of 18°C to allow efficient activity of the *Gal4* drivers, except for the chromosome in Figure 3A, which was obtained at 18°C in order to improve cytology of the chromosomes. RNase A treatment of polytene chromosomes squashes was performed as described in [60]. Rabbit anti-Topo I (1/400) antibodies were kindly provided by Tao Hsieh. Anti-Pol II mAb H14 was purchased (Covance). To detect GFP-Topo I on squashed polytene preparations, we used a rabbit anti-GFP antibody (Invitrogen, used at 1/200), because GFP fluorescence is destroyed by the fixation method employed. The anti-B52 serum was raised in rats by Eurogentec against the sequence KNGNASPDRNNESMDD, which corresponds to the last 16 amino acids of B52. This serum was purified with the Melon Gel IgG Purification Kit (Pierce). This serum recognizes a single polypeptide of 52 kDa in western blots, corresponding to B52 (Figure 2C and 4B, panel B52), moreover no signal is detected with this serum on B52 mutant chromosomes (not shown) or on chromosomes from B52-depleted larvae (Figure 4F), indicating that the staining is specific for B52. It recognizes the same band on western and on polytene chromosomes as a rabbit serum raised against the same peptide [21]. The purified rat anti-B52 antibody was used at 1/50 dilution on squashed polytene chromosomes. Secondary antibodies from Molecular Probes were used at a dilution of 1/400 (anti-rat-Alexa546, anti-rabbit-Alexa488, anti-mouse IgM-Alexa488, anti-rabbit-Cy5). For staining of larval imaginal discs, anti-Topo I antibody was used at 1/2000. For staining of intact salivary glands, anti-B52 was used at 1/500, anti-Fibrillarin monoclonal antibody 72B9 was used at 1/100, and corresponding secondary antibodies were used at 1/3000. Tissues or polytene chromosomes were all stained with Hoechst and mounted in Prolong Gold Antifade (Molecular Probes). Images were acquired on a Zeiss AxioimagerZ1 drived by Metamorph for conventional fluorescence, or by Axiovision for acquisition with Apotome. Overlay images were reconstituted in Adobe Photoshop software.

### *hsp70* expression analysis

For RNA-FISH analysis, third instar larvae were heat shocked during 20 min at 37°C in a water bath. To prepare the RNA probes, a 1.1 kb PCR fragment corresponding to the 5′ half of *hsp70* gene was cloned in pCR II vector (Invitrogen). This plasmid was linearized by restriction enzyme digestion and *in vitro* transcribed with SP6 or T7 RNA polymerase (Biolabs) in the presence of digoxigenin-labeled UTP (Roche Diagnostics) to produce antisense or sense probe respectively. The probes were purified with RNeasy kit (Qiagen). *In situ* hybridization was performed as described in [61] with 50 ng of RNA probe. Following hybridization, RNA probes were detected with mouse anti-DIG antibodies (1/500, Roche Diagnostics). B52 and Topo I proteins were detected with purified rabbit anti-B52 (1/500) and rabbit anti-Topo I (1/2000) antibodies respectively. Secondary antibodies (anti-mouse-Alexa555 and anti-rabbit-Alexa488 from Molecular Probes) were used at 1/4000. Hybridization with the sense probe gave no signal, as expected (not shown).

To prepare Northern blots, total RNA was extracted from wild type or mutant larvae with tri-reagent (Sigma), and quantified with Nanodrop. Mutant larvae (*B52* ^S2249^, *B52* ^S2249^/*B52* ^28^, and *top1* ^77^ were heat shocked at the second instar, whereas we used third instar larvae for RNAi mediated depletion. The larvae were heat shocked during 40 min at 37° and allowed to recover between 0 to 6 h at 25°C. 5 µg of total RNA was loaded on a 1.3% agarose gel containing 6% formaldehyde. RNAs were transferred to nitrocellulose membrane in 10X SSC and then cross-linked by baking the membrane 3 h at 80°C. The plasmid containing the 1.1 kb PCR fragment corresponding to the 5′ half of *hsp70* gene was used to prepare the *hsp70* probe. The probes were prepared by random priming in the presence of ^33^P-dCTP and purified with NucleoSpin Extract II kit (Macherey-Nagel). Hybridization was performed overnight at 42°C. Radiactive signals were detected by phosphor-imager.

For western blots, HSP70 protein was detected with a monoclonal anti-HSP70 (5A5, Thermo Scientific) and ECL detection (Pierce).

### Chromatin immunoprecipitation

Wild type and *B52* RNAi third instar larvae were homogenized with dounce in fixation solution buffer A (50 mM Hepes, 1 mM EDTA, 0.5 mM EGTA, 15 mM NaCl, 60 mM KCl, 0.1% tritonX-100 and Protease inhibitor cocktail SetI (Calbiochem)) containing 1.8% formaldehyde. Homogenates were filtered through 100 µm filters. After 12 min fixation, glycine was added to 0.225 M. Nuclei were pelleted by centrifugation, washed twice in buffer A, and finally resuspended in buffer B (50 mM Hepes, 1 mM EDTA, 1% NP40, 0.1% SDS, 0.1% NaDeoxycholate and Protease inhibitor cocktail SetI (Calbiochem)) containing 140 mM NaCl. Nuclei were sonicated 10 times 30 sec on ice with a Vibra-Cell ultrasonic processor, at amplitude 50. Debris were pelleted by centrifugation 5 min at 16000 g. Soluble chromatin was pre-cleared 1 h with magnetic protein-G Dynabeads (Invitrogen) at 4°C. For immunoprecipitation, chromatin corresponding to approximately 10 larvae was incubated overnight at 4°C with anti-RNA Pol II antibody (8WG16, 4 µl) or no antibody (Mock) in the presence of 30 µl protein-G Dynabeads. Beads were washed at room temperature in buffer B containing 140 mM NaCl (3×5 min), then 300 mM NaCl (3×5 min), then 250 mM LiCl (2×5 min), and finally in TE (2×5 min). Elution from the beads was performed at 65°C with 300 µl elution buffer (100 mM NaHCO3, 1% SDS) and shacking. To reverse the crosslinks, NaCl was added to 300 mM and the tubes were incubated 7 h at 65°C. After RNase A and proteinase K treatments, DNA was purified by phenol-chloroform extraction and ethanol precipitation, and finally dissolved in 60 µl H_2_O.

### Quantitative PCR analysis

Real-time PCR was performed using SYBR Green and the LightCycler 480 real-time PCR system (ROCHE), in 10 µl with 2.5 µl purified DNA per reactions. PCR settings: 2 min 95°C; 45 cycles: 10 s 95°C, 15 s 68°C, 25 s 72°C. Data were collected at 72°C. PCR were performed in triplicates on two independent immunoprecipitations. For each PCR and each chromatin preparation, a standard curve is made on purified input DNA (purified from aliquots of chromatin taken before immunoprecipitation). The amount of target sequence in immunoprecipitated DNA samples was expressed as a percentage of DNA present in the input material. We used primers for *hsp70* sequences centered at −154 (from −200 to −108), +378 (from 334 to 423), +1426 (from 1363 to 1490) and +1951 (from 1925 to 1978), as described by Boehm *et al*. [37], [62]. Primers located downstream from the *hsp70* gene in the *Octβ3R* gene [37], [63] are used as a negative control.

## Supporting Information

Figure S1 Polytene chromosomes from *Wee-P153* larvae, triple stained with anti-B52, anti-GFP (revealing GFP-Topo I) and anti-Pol II antibodies. The right panel shows composite images of B52 and Topo I staining or B52 and Pol II staining, presented as overlay or split along half of each chromosome.
(1.59 MB TIF)

Figure S2 Immunodetection of B52 and Topo I on polytene chromosomes from control (*sgs3-gal4/+*) or Topo I-overexpressing (*sgs3-gal4/UAS-Topo I#11*) salivary glands. Overexpression of Topo I enhances the banding pattern detected with the Topo I antibody, as faint bands become strongly visible upon Topo I overexpression. Note, Topo I staining coincides with stronger B52 signal.
(0.94 MB TIF)

Figure S3 (A) Immunodetection of B52 and GFP-Topo I on polytene chromosome from salivary glands of the *WeeP-153* line, overexpressing B52 (genotype: *y,w,WeeP-153/Y; sgs3-gal4/+; UAS-B52/+*). (B) Western blot analysis of control (genotype: *sgs3-gal4/+*) or B52-overexpressing (B52 OE, genotype: *sgs3-gal4/+; UAS-B52/+*) salivary gland extracts.
(0.74 MB TIF)

Figure S4 Fragments of polytene chromosomes surrounding the insertion point of the *UAS-BBS(5.12)* transgene (arrowhead), in the absence (A) or presence (B) of the driver *sgs3-gal4*. Chromosomes are shown stained with anti-B52 and anti-GFP antibodies (the latter detects GFP-TopoI). In the presence of GAL4, expression of *UAS-BBS(5.12)* is strongly activated, creating a puff at the transgene insertion site (B). Both B52 and TopoI are strongly recruited to this site. Genotypes: (A) *y,w,WeeP-153/Y; UAS-BBS(5.12)/+* and (B) *y,w,WeeP-153/Y; UAS-BBS(5.12)/sgs3-gal4*.
(1.14 MB TIF)

Figure S5 Immunostaining of salivary glands from control (*y,w,WeeP-153/Y; da-gal4/+*) or B52-depleted larvae (*y,w,WeeP-153/Y; da-gal4/UAS-IR-B52*), with anti-B52 and anti-fibrillarin antibodies. GFP-Topo I is detected by the intrinsic fluorescence of GFP.
(2.55 MB TIF)
