# Supplementary figures and images for: The SR Protein B52/SRp55 Is Required for DNA Topoisomerase I Recruitment to Chromatin, mRNA Release and Transcription Shutdown
Source: PLoS Genet. 2010 Sep 16;6(9):e1001124. doi: 10.1371/journal.pgen.1001124 (PMC2940736; doi:10.1371/journal.pgen.1001124)

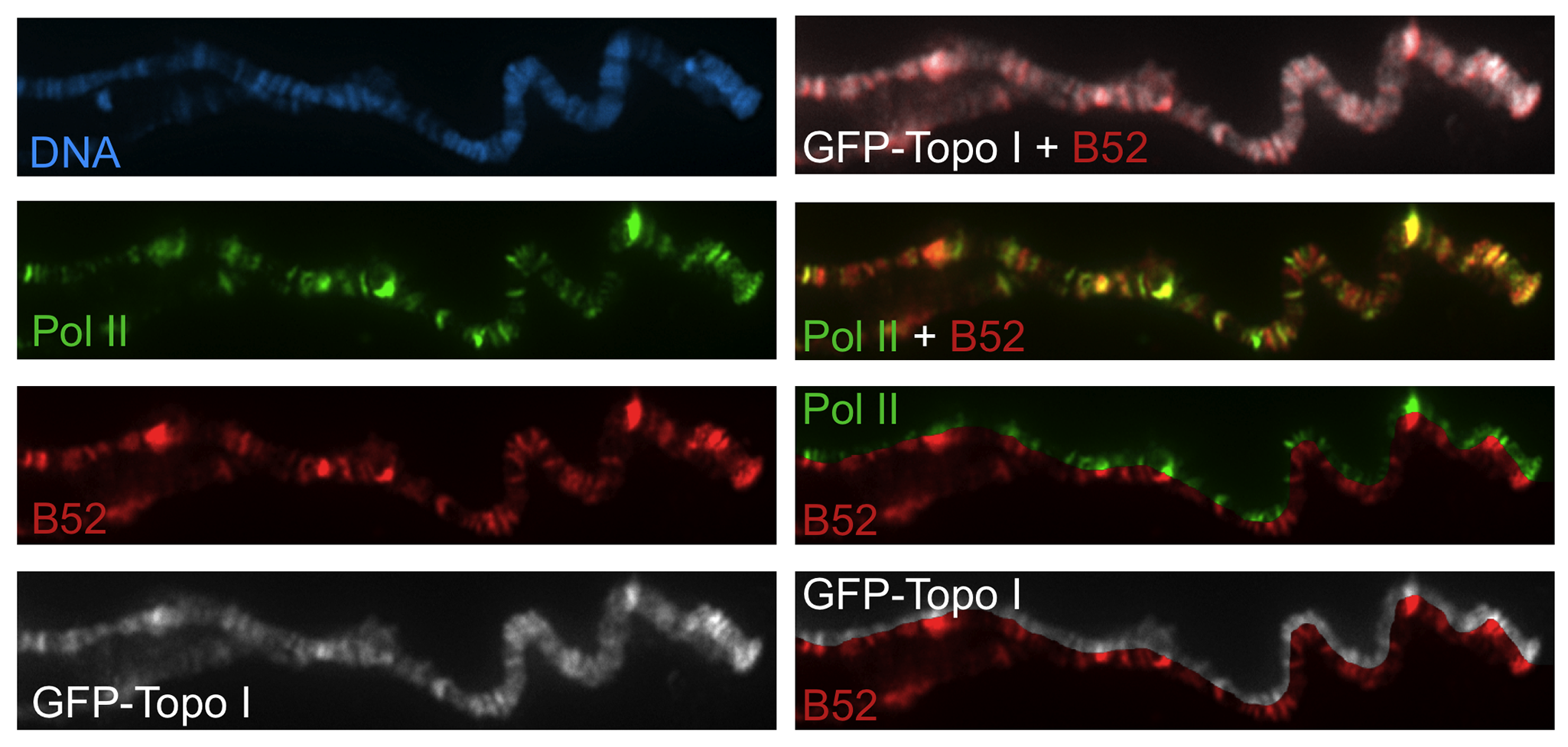

Supplement: Figure S1 — Polytene chromosomes from Wee-P153 larvae, triple stained with anti-B52, anti-GFP (revealing GFP-Topo I) and anti-Pol II antibodies. The right panel shows composite images of B52 and Topo I staining or B52 and Pol II staining, presented as overlay or split along half of each chromosome. (1.59 MB TIF) [file pgen.1001124.s001.tif]

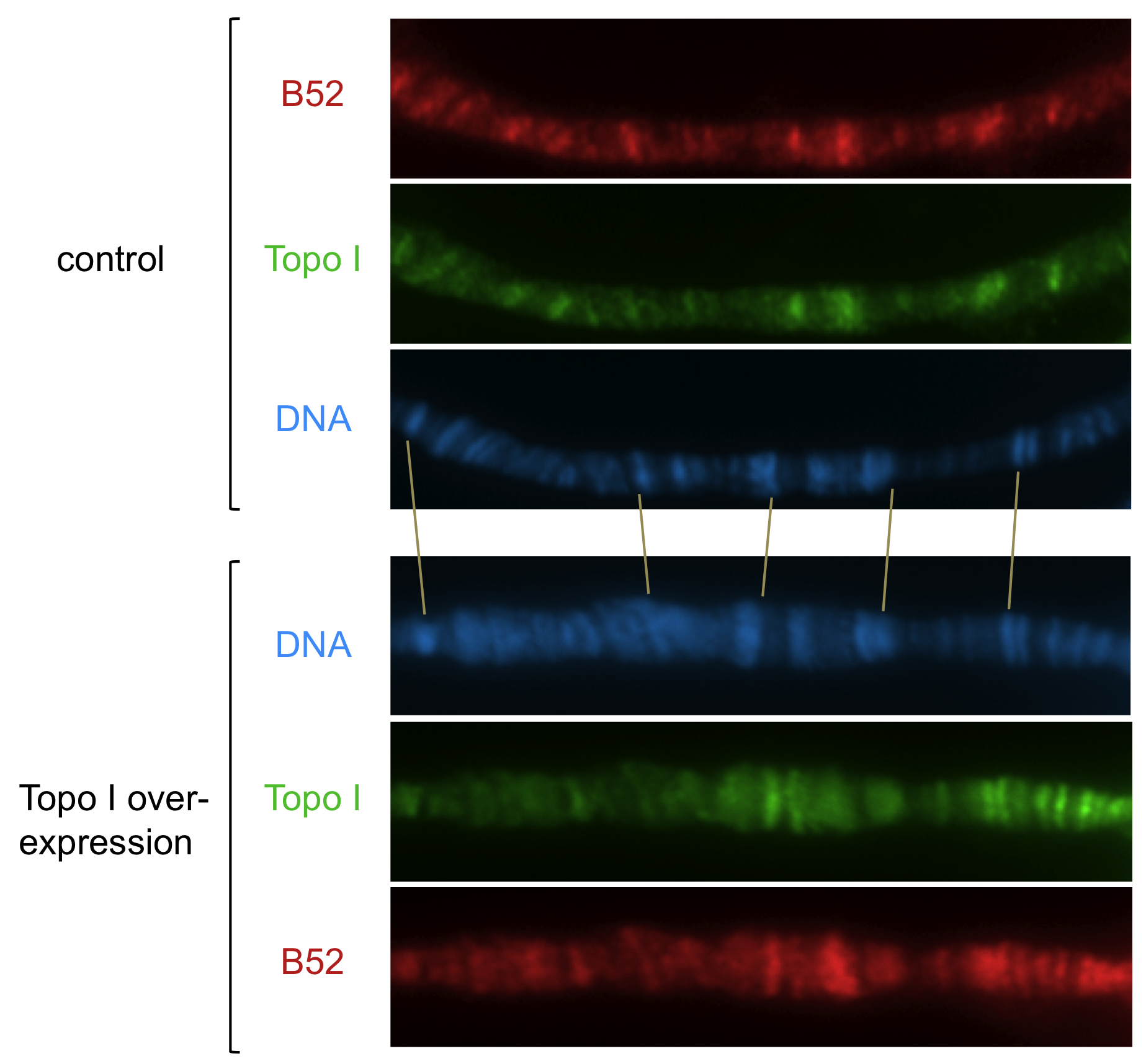

Supplement: Figure S2 — Immunodetection of B52 and Topo I on polytene chromosomes from control (sgs3-gal4/+) or Topo I-overexpressing (sgs3-gal4/UAS-Topo I#11) salivary glands. Overexpression of Topo I enhances the banding pattern detected with the Topo I antibody, as faint bands become strongly visible upon Topo I overexpression. Note, Topo I staining coincides with stronger B52 signal. (0.94 MB TIF) [file pgen.1001124.s002.tif]

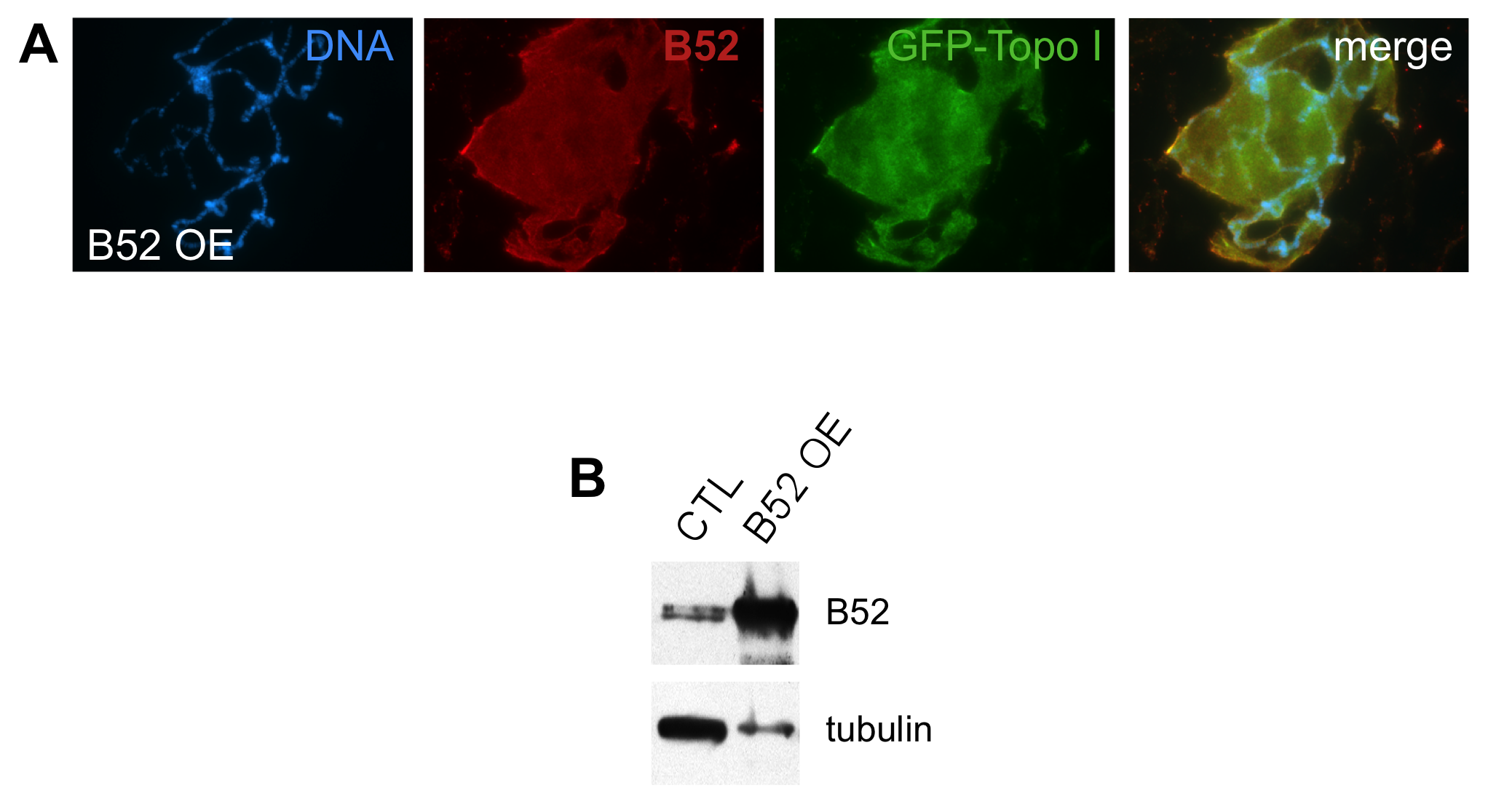

Supplement: Figure S3 — (A) Immunodetection of B52 and GFP-Topo I on polytene chromosome from salivary glands of the WeeP-153 line, overexpressing B52 (genotype: y,w,WeeP-153/Y; sgs3-gal4/+; UAS-B52/+). (B) Western blot analysis of control (genotype: sgs3-gal4/+) or B52-overexpressing (B52 OE, genotype: sgs3-gal4/+; UAS-B52/+) salivary gland extracts. (0.74 MB TIF) [file pgen.1001124.s003.tif]

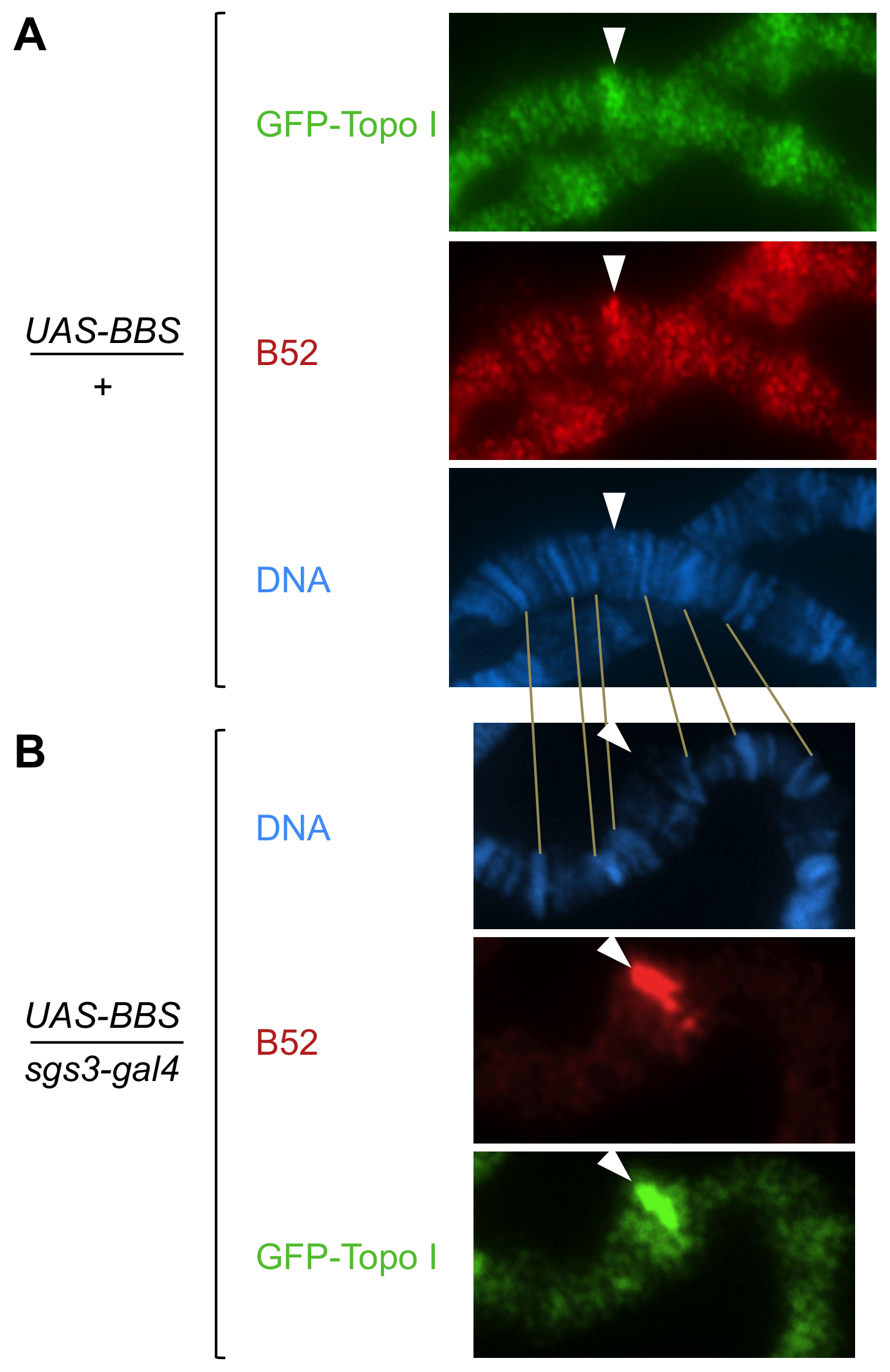

Supplement: Figure S4 — Fragments of polytene chromosomes surrounding the insertion point of the UAS-BBS(5.12) transgene (arrowhead), in the absence (A) or presence (B) of the driver sgs3-gal4. Chromosomes are shown stained with anti-B52 and anti-GFP antibodies (the latter detects GFP-TopoI). In the presence of GAL4, expression of UAS-BBS(5.12) is strongly activated, creating a puff at the transgene insertion site (B). Both B52 and TopoI are strongly recruited to this site. Genotypes: (A) y,w,WeeP-153/Y; UAS-BBS(5.12)/+ and (B) y,w,WeeP-153/Y; UAS-BBS(5.12)/sgs3-gal4. (1.14 MB TIF) [file pgen.1001124.s004.tif]

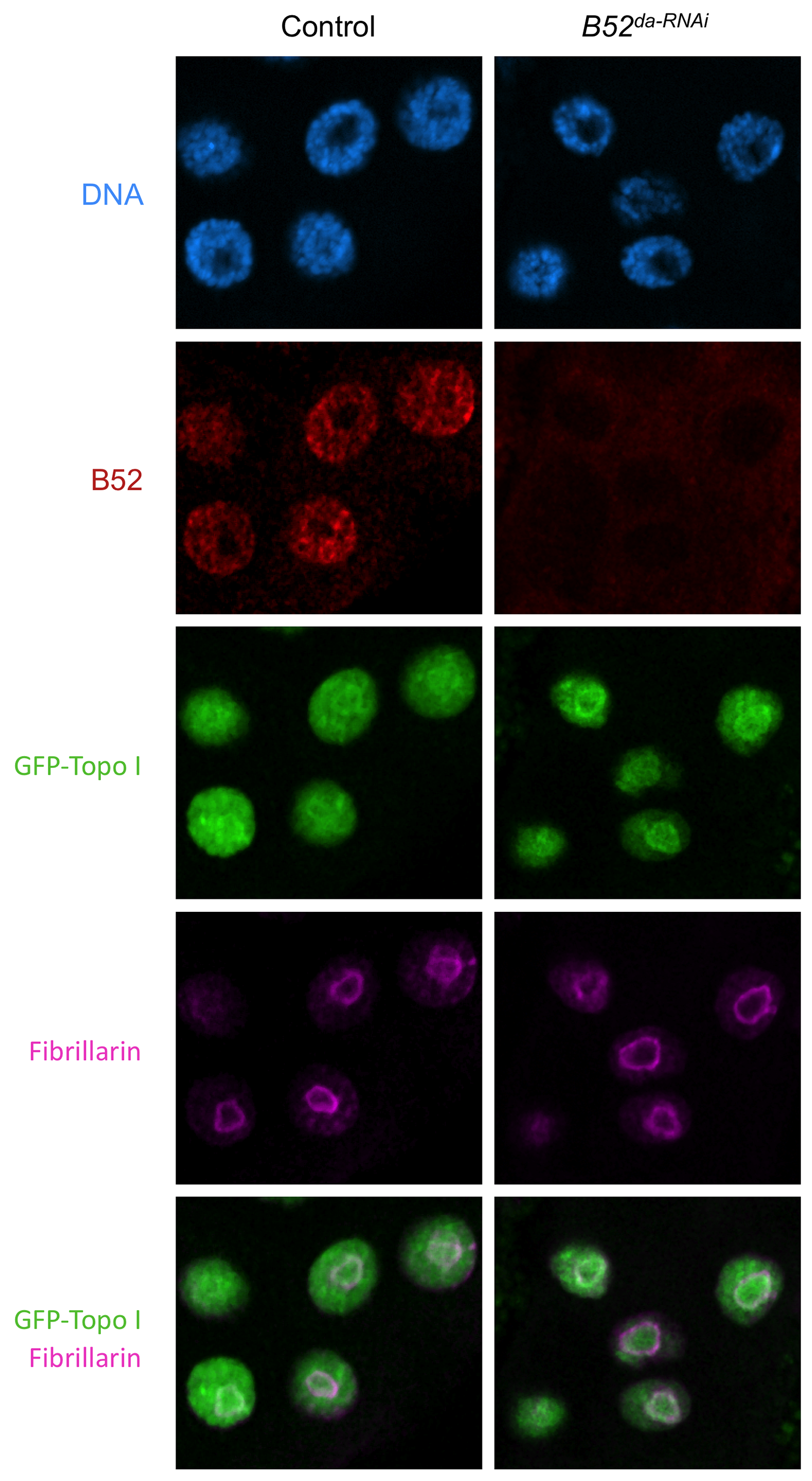

Supplement: Figure S5 — Immunostaining of salivary glands from control (y,w,WeeP-153/Y; da-gal4/+) or B52-depleted larvae (y,w,WeeP-153/Y; da-gal4/UAS-IR-B52), with anti-B52 and anti-fibrillarin antibodies. GFP-Topo I is detected by the intrinsic fluorescence of GFP. (2.55 MB TIF) [file pgen.1001124.s005.tif]
